# GDSL Lipases/Esterases: Versatile Regulators of Plant Development and Stress Resilience

**DOI:** 10.3390/ijms27093872

**Published:** 2026-04-27

**Authors:** Ke Dong, Rehman Sarwar, Yuanxue Liang, Wei Zhang, Rui Geng, Wenlong Jiang, Xiang Fan, Xiao-Li Tan

**Affiliations:** 1 School of Life Sciences, Jiangsu University, Zhenjiang 212013, China; 2212317028@stmail.ujs.edu.cn (K.D.); rehman_sarwar@outlook.com (R.S.); yuanxue@ujs.edu.cn (Y.L.); weizhang@ujs.edu.cn (W.Z.); 2112218001@stmail.ujs.edu.cn (R.G.); koi_loong@163.com (W.J.); 2212317035@stmail.ujs.edu.cn (X.F.); 2 School of Agricultural Engineering, Jiangsu University, Zhenjiang 212013, China

**Keywords:** GELP family, lipid metabolism, stress response, functional genomics

## Abstract

GDSL esterase/lipase (GELP) proteins constitute an evolutionarily conserved yet functionally diversified hydrolase family in land plants. They participate in cuticle and secondary cell wall biosynthesis, seed lipid remobilization, reproductive development, and hormone-mediated responses to biotic and abiotic stresses. Despite extensive genome-wide and comparative genomic studies that have categorized large GELPs across numerous crops and model species, only a fraction of members have been functionally characterized in plants, and their catalytic mechanisms and regulatory architectures remain poorly understood. Recent population genomics and cross-species orthogroup analyses in 46 angiosperms have uncovered substantial natural variation within GELP coding sequences and regulatory regions, providing a powerful framework to link allelic diversity to evolutionary trajectories and physiological functions. This review synthesizes current knowledge on GELP evolution, biochemical properties, and roles in development and stress adaptation, and critically evaluates how these insights can be translated into biotechnology and molecular breeding strategies. It highlights emerging resources and concepts from orthogroup-based classification and multi-species datasets that enable systematic discovery of GELP alleles affecting agronomic traits. It further outlines research exploiting GELPs in crop improvement, emphasizing the integration of reverse and forward genetics with multi-omics profiling, biochemical and structural characterization, and gene regulatory network reconstruction. Systematic assessment of the phenotypic impacts of single and combinatorial GELP perturbations on yield, quality, and stress resilience is proposed as a key step toward translating basic insights into breeding and engineering strategies.

## 1. Introduction

GDSL esterase/lipases/acyltransferases (GELPs) constitute a large and widely distributed family of lipolytic enzymes occurring in prokaryotes and eukaryotes and performing diverse biological functions, particularly in plants [1,2,3]. These enzymes are defined by an N-terminal GDSL (GDSxxDxG) motif and a conserved catalytic architecture comprising four invariant residues, Ser, Gly, Asn, and His located in five consensus blocks (I–V), which places them within the SGNH hydrolase superfamily [4]. The active-site Ser together with Asp, Glu, and His forms a catalytic triad, whereas Gly and Asn contribute to the oxyanion hole, providing a structurally flexible active site that underlies the broad substrate specificity and stereoselectivity typical of GELPs [2,5]. In contrast to classical lipid substrates, such as thioesters, aryl esters, phospholipids, and lysophospholipids, many GELPs hydrolyze a variety of ester and amide bonds and exhibit thioesterase, arylesterase, and protease-like activities [4,5]. Moreover, plant-derived GELPs such as those involved in pyrethrin modification and secondary metabolite processing function as acyltransferases, catalyzing esterification reactions in specialized metabolic pathways [6], thereby extending the catalytic repertoire of the family beyond simple hydrolysis.

In plants, GELPs form one of the largest lipase gene families, typically encompassing from several dozen to a few hundred genes per genome. Genome-wide analyses have identified 105, 114, 194, and 121 GELP genes in *Arabidopsis thaliana* (*A. thaliana*), *Oryza sativa* (*O. sativa*), *Glycine max* (*G. max*), and *Brassica rapa* (*B. rapa*), respectively, and 83–130 members in *Vitis vinifera* (*V. vinifera*), *Populus* spp., and *Sorghum bicolor* [4]. More extensive families have been reported in polyploid crops, including 193 GELPs in *Dasypyrum villosum* (*D. villosum*), 240 in *Brassica napus* (*B. napus*), nearly 200 in *Gossypium* species, and over 500 in *Triticum aestivum* (*T. aestivum*), reflecting lineage-specific expansions driven mainly by whole-genome and tandem duplications [7]. Despite these advances, only a small fraction of plant GELPs have been functionally characterized. The structural plasticity of their binding pockets, while enabling promiscuous activity, complicates the identification of in vivo substrates and pathways [8]. Understanding the physiological and molecular functions of plant GELPs is essential for precise functional annotation of this large gene family and for exploiting GELP alleles as targets in crop improvement. Insights into how individual GELPs regulate cuticle and secondary wall formation, male fertility, seed lipid mobilization, hormone signaling, and tolerance to drought, salinity, heat, cold, and pathogens provide concrete entry points for breeding and engineering crops with stacked resistance and tolerance traits [6,7]. As climate change intensifies combined biotic and abiotic stresses, GELP-based biotechnological and breeding strategies offer promising opportunities to enhance yield stability and food security. In this review, recent advances in the identification, evolution, and biochemical activities of plant GELPs are synthesized, with particular emphasis on their roles in plant growth, development and plant–environment interactions, and on the emerging applications of GELPs in molecular breeding for resilient crops.

## 2. Evolutionary Insights into the GELPS Domains

Phylogenomic classification across 46 flowering plants resolves GELPs into 10 main clusters and 44 orthogroups, highlighting highly conserved catalytic cores but contrasting evolutionary dynamics, with some orthogroups maintained as single-copy and others showing massive monocot, dicot, or wheat-specific amplification [9]. Consistent with earlier work, most plant GELP genes retain a characteristic 5-exon, 4-intron structure, yet recent studies emphasize clade-specific intron gain, and motif turnover, especially in *G. max*, and *Gossypium hirsutum* (*G. hirsutum*), where large subfamilies display extensive exon-intron and motif variation [2,7,10]. Comparative genomics and synteny analyses across *G. max*, *B. napus*, *G. hirsutum*, *D. villosum*, and *Malus domestica* indicate that whole-genome/segmental duplication plus tandem duplication are the major drivers of family expansion, while the Ka/Ks ratio (the ratio of nonsynonymous to synonymous substitution rates), ranging from 0.1 to 0.3 for most duplicated gene pairs, supports pervasive purifying selection on the SGNH catalytic scaffold [2,7,8]. Beyond copy number growth, recent 3D modeling and docking in *G. hirsutum* show that divergence of binding pocket architecture and electrostatics, rather than core fold disruption, underpins substrate and functional diversification among paralogs. Expression atlases across tissues, developmental stages, and stress conditions in *G. max*, *B. napus*, *D. villosum*, *G. hirsutum*, and *Citrullus lanatus* (*C. lanatus*) reveal broad functional diversification of GELPs [3,8,11,12]. Discrete GELP subsets are preferentially associated with pollen and another development, fruit and fiber formation, seed germination and seedling establishment, and cell wall and cuticle biogenesis. Other subsets contribute to lipid mobilization and hormone-regulated responses to drought, salinity, cold, pathogens, and viruses.

Collectively, current genomic, structural, and transcriptomic data reinforce GELPs as a rapidly expanded yet structurally constrained gene family in which duplication-driven, binding-pocket-centered diversification around a conserved SGNH core supports key roles in plant development and environmental adaptation. Importantly, orthogroup-resolved repertoires and synteny maps now provide practical resources for identifying conserved GELP loci associated with agronomic traits and for transferring functional annotations across crops, thereby facilitating marker development and candidate-gene selection in breeding programs.

## 3. Roles of GELPS in Plant Development and Plant Metabolism

### 3.1. The Multifaceted GDSL-Type Esterase Lipases in Plant Reproductive Development

**Figure 1:**
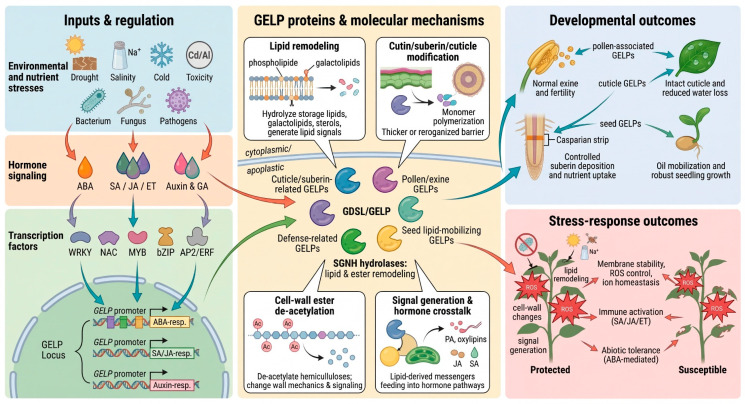
Molecular functions of GDSL esterase/lipases (GELPs) in plant responses to environmental stresses. GELPs integrate lipid remodeling, hormone signaling (abscisic acid, jasmonic acid, ethylene, salicylic acid, and gibberellin), and reactive oxygen species (ROS) homeostasis to support membrane stability, cuticle and secondary wall formation, and growth–defense balance under abiotic and biotic stresses. ABA, abscisic acid; JA, jasmonic acid; ET, ethylene; SA, salicylic acid; GA, gibberellin; ROS, reactive oxygen species. This figure is created using BioRender.com.

GDSL exhibits broad functional diversity in development and stress responses, with a growing subset shown to be central to male fertility [1,4,9,13]. Multiple GELPs are now functionally linked to anther and pollen development in diverse species: *EXL4* and *EXL6* in *A. thaliana* and their *B. rapa* ortholog *BrEXL6*, as well as *BrGGL7*, are required for normal pollen formation and for generating pollen coat lipids that mediate efficient pollen stigma interaction and hydration [1,14,15]. In cereals, *ZmMs30* in *Zea mays* (*Z. mays*) and *OsGELP34*, *OsGELP110*, and *OsGELP115* in *O. sativa* are essential for anther cuticle integrity and pollen exine biogenesis, and loss-of-function alleles cause complete or near-complete male sterility [4,16,17]. *RMS2*, an endoplasmic reticulum-localized GDSL lipase in *O. sativa*, provides a mechanistic example in which a mutation within the GDSL domain reduces hydrolase activity and alters the abundance of at least 16 lipid species, disrupting tapetum degradation, anther cuticle formation, and pollen wall development, thereby compromising fertility [16]. In *A. thaliana*, *GELP77* is indispensable for pollen dissociation and surface patterning, with mutants producing shrunken, aggregated pollen grains and severe reproductive defects, while *TaGELP073* knockdown in *T. aestivum* similarly impairs anther and pollen development and reduces fertility [9,18,19] (Figure 1).

Genome-wide orthogroup classification has placed these fertility-related genes into *OG-GELP-C7a*, *C8f*, *C10a*, *C10b*, and *C10h*, supporting the view that distinct stages of pollen development rely on specific lipid-modifying reactions catalyzed by different GELPs [9]. However, phylogenetically close genes can participate in divergent processes, and conversely, phylogenetically distant GELPs may converge on similar reproductive functions, indicating substantial sub-functionalization and neofunctionalization within this family [8]. Despite clear genetic evidence that many GELPs act as key nodes in lipid metabolism underlying anther cuticle, pollen wall, and pollen coat formation, the precise natural substrates, reaction specificities, and pathway contexts remain poorly defined for most enzymes [7,20]. Addressing this gap will require systematic integration of targeted metabolomic and lipidomic profiling of reproductive tissues from GELP mutants, structural and binding-pocket analyses, and high-resolution imaging of anther and pollen architecture, combined with reverse genetics and multi-omics approaches, to mechanistically resolve how individual GELPs orchestrate lipid-based signaling and structural remodeling during plant reproduction. From an applied perspective, male-sterile *gelp* alleles and conditional fertility represent promising tools for molecular breeding, for example, in three-line and two-line hybrid seed production systems in *O. sativa*, *T. aestivum*, and *Brassica* crops. Precise editing and introgression of GELP loci controlling anther cuticle formation and pollen exine can be strategically used to generate stable cytoplasmic- or nuclear-based male sterility, thereby enabling efficient hybrid seed production and heterosis exploitation.

### 3.2. GELPS in Pollination and Early Pollen Stigma Interactions

In species with dry stigmas, compatible pollination involves local loosening and disruption of the stigmatic cuticle by cutinase-like lipases, making the identification of such plant cutinases important for understanding the molecular basis of pollen-stigma interactions [1,2,21]. In *A. thaliana*, the GDSL enzyme *CDEF1* is expressed in pollen and pollen tubes and localizes to the pollen stigma interface, and biochemical work links it to cutin-related substrates, although its precise in planta function during pollination remains unresolved. Pollination on dry stigmas is initiated by pollen hydration, a tightly regulated process that depends on lipids and proteins in the pollen coat and stigmatic papilla surface [14,22]. On the pollen side, the extracellular GDSL-like lipase *EXL4* is required for efficient hydration, as *exl4* mutants show delayed water uptake; *EXL4* acts together with the pollen-coat oleosin-domain protein *GRP17* to promote the initiation and progression of hydration [7,23]. Complementing these pollen-derived factors, stigmatic receptor kinases such as *SD-RLK28* and members of the *RKF1* and *RKFL1* cluster, as well as papilla vacuole dynamics and ROS signaling, have recently emerged as additional regulators that control water release from papilla cells to compatible pollen grains [21,23,24] (Figure 1). These findings suggest that GELPs at the pollen-stigma interface could be manipulated to fine-tune self-compatibility, cross-compatibility, and fertilization efficiency. For instance, targeted modification of GELP-dependent pollen coat properties or stigmatic cuticle remodeling might be harnessed in biotechnology to control gene flow, manage reproductive barriers between wild relatives and crops, and improve pollination success under suboptimal environmental conditions.

### 3.3. GELPs in Fruit and Seed Development

Comparative genomic analyses in *Rosaceae* have identified distinct GELP subfamilies that are preferentially expressed during fruit development, particularly in pericarp and developing *Pyrus communis* fruits, suggesting that specific GELP subsets contribute to fruit surface formation and ripening-related processes [1,10,25]. In *S. lycopersicum*, mutations in surface-localized GELPs, such as *SlGDSL1*, *SlGDSL2*, and the cutin synthase *SlCUS1*, and *SlCD1* primarily alter cuticle architecture and surface gloss, while having little effect on overall fruit size, consistent with the broader *SlGELP* family analysis that links many *S. lycopersicum* GELPs to epidermal and stress-related roles rather than bulk growth [1,26]. By contrast, seed traits are highly responsive to some GELP genes. In *B. napus*, overexpression of the sinapine esterase *BnSCE3* and *BnLIP2* under a seed-specific promoter drastically reduces seed sinapine levels and increases choline, which feeds into phospholipid metabolism, and this metabolic shift is accompanied by larger seeds, higher 1000-kernel weight, and increased seed water content [1,27]. Similarly, ectopic expression of the *G. hirsutum* GDSL lipase *GhGLIP* in *A. thaliana* enhances seed length and fresh weight and is associated with greater soluble sugar and protein accumulation, indicating a positive role in seed growth [28,29] (Table 1). In *A. thaliana*, the bHLH transcription factor retarded growth of Embryo1 (*RGE1*, acts from the endosperm to control embryo growth; *rge* loss-of-function mutants produce small, shriveled seeds and show strong down-regulation of two GDSL-motif lipase genes, whereas single mutants of these GELPs lack obvious seed defects, pointing to functional redundancy within the GELP family and integration of GELP-mediated processes into broader seed-development transcriptional networks [1,30,31] (Table 1). Together, these studies reveal several biotechnology-relevant entry points. For instance, seed-associated GELPs can be targeted to improve oil quality and reduce anti-nutritional compounds, whereas cuticle-associated GELPs can be used to enhance fruit shelf-life and stress resistance. Incorporating GELP alleles into marker-assisted selection pipelines and editing promoter regions to adjust tissue-specific expression offer practical strategies for tailoring quality traits while minimizing pleiotropic effects.

### 3.4. GELPs in Secondary Metabolism and Xenobiotic Processing

Several GELPs have been recruited into specialized metabolic pathways. In *Rauvolfia verticillata*, a pathway-specific 17-O-acetylnorajmaline esterase (AAE) hydrolyzes 17-O-acetylnorajmaline and related 17-O-acetylajmalan intermediates to their deacetylated forms, constituting one of the terminal acyl-removal steps in ajmaline biosynthesis [40,41,42]. In *Digitalis lanata*, a cell-wall-localized lanatoside-15′-O-acetylesterase (LAE) deacetylates 15′-O-acetylated lanatoside cardenolides to the corresponding purpureaglycosides, thereby remodeling cardenolide glycoside profiles in the plant [43,44]. Recent completion of the ajmaline pathway and identification of sterol-cleaving CYP87A enzymes that form pregnenolone in cardenolide biosynthesis underscore that such esterases operate within well-defined, but still expanding, biosynthetic networks [40,45].

Phylogenetically related GELPs act on other small esters: plant acetylcholinesterase homologs from *Macroptilium atropurpureum*, *Salicornia europaea*, and *Z. mays* hydrolyze acetylthiocholine and propionylthiocholine, whereas the *A. thaliana* ortholog *At3g26430* lacks acetylthiocholine activity and instead prefers longer-chain acyl substrates, illustrating pronounced functional divergence within a single *GELP* orthogroup [32]. Beyond endogenous metabolites, GELPs can target xenobiotics; for example, the black-grass (*Alopecurus myosuroides*) enzyme GDSH1 hydrolyzes aryloxyphenoxypropionate herbicide esters and bioactivates them, impacting herbicide efficacy against this weed [1]. Recent work further broadens this view by showing that GELPs such as *TcGLIP* in pyrethrin biosynthesis, *S. lycopersicum SlCGT* and *Sl-LIP8* in hydroxycinnamate and volatile production, and *Cichorium intybus CiCQE1*, and *CiCQE3* in chlorogenic-acid remobilization operate as hydrolases or acyltransferases in diverse secondary metabolic branches, while AChE-like GELPs in *S. lycopersicum* and other species are increasingly linked to abiotic stress responses within an emerging Ach-AChE signaling framework [1,8,46,47,48].

The recruitment of GELPs into specialized and xenobiotic metabolism opens clear avenues for biotechnology. GELPs involved in the biosynthesis and modification of pharmacologically active alkaloids, cardenolides, and pyrethrins can be used to engineer metabolic flux toward high-value compounds in medicinal plants or heterologous production systems. Conversely, GELPs such as *GDSH1* that bioactivate herbicides highlight potential targets for managing herbicide resistance in weeds through chemical design and gene-based diagnostics. More broadly, the substrate promiscuity and acyltransferase activities of GELPs can be exploited in synthetic biology to construct novel esterification pathways, design tailor-made biocatalysts for green chemistry, and generate new-to-nature molecules with agronomic or industrial utility.

## 4. Function of GELPs in Plant Stress Responses

Extensive studies across multiple species have demonstrated that many GELPs are inducible by diverse abiotic and biotic stresses, supporting their roles in plant defense and stress adaptation. Clarifying the precise molecular functions of GELPs and how they integrate signaling networks underlying plant responses to combined abiotic and biotic stresses will facilitate the breeding and engineering of more resilient crops suited to rapidly changing climates [1,2,7,13]. Defined by an N-terminal motif, GELPs exhibit broad substrate and positional specificity, enabling the hydrolysis and remodeling of structurally diverse lipid species [1,4,37]. Accumulating evidence now supports an emerging view that at least a subset of GELPs function as integrators of specialized lipid metabolism, development, and environmental acclimation, although most family members remain functionally uncharacterized [1,2,7,49]. Functionally, GELP-mediated lipolytic activities contribute to membrane remodeling, production of lipid-derived signaling molecules, modulation of salicylic acid (SA), jasmonic acid (JA), ethylene (ET), abscisic acid (ABA), gibberellin (GA) and auxin pathways, and the synthesis or turnover of cuticle, wax and suberin barriers, thereby supporting cellular homeostasis and growth–defense balance under stress [1,7,19,49]. Genome-wide expression and multi-omics datasets, together with reverse genetics and biochemical assays, are beginning to pinpoint GELP isoforms that can be exploited through genome editing and breeding to improve multi-stress tolerance in crops (Table 1). Notably, several *G. max*, *Sedum alfredii* (*S. alfredii*), *Lilium pumilum* (*L. pumilum*), *Ricinus communis* (*R. communis*), *G. hirsutum*, and *B. napus* GELPs have been functionally linked to drought, salinity, heavy-metal, and temperature tolerance, providing proof-of-concept that GELP alleles can serve as practicable targets for enhancing stress resilience in agriculture.

Recent genome-scale studies underscore the breadth and plasticity of GELP repertoires across species [1,14,37]. In *R. communis*, 96 *RcGELP* genes have been identified and annotated, their promoters enriched in hormone and stress-responsive *cis*-elements. Transcriptomic profiling during germination and early seedling establishment shows that many *RcGELPs* are strongly induced or repressed by high temperature and salt, forming dynamic regulatory clusters associated with early stress adaptation [19]. Although direct biochemical and genetic validation of *RcGELP* function is still lacking, this coordinated, stress-responsive expression pattern, together with promoter motif composition, supports the hypothesis that *RcGELPs* contribute to lipid metabolism and possibly membrane-associated processes during critical developmental stages. Similar genome-scale and functional studies in other crops, where individual GELPs enhance drought, salt, heat tolerance or reproductive development, further strengthen this functional framework for *RcGELPs* [4,13,37,50].

By contrast, *L. pumilum LpGDSL* exemplifies a GELP whose stress-related function and molecular context are resolved at much higher resolution. *LpGDSL* is rapidly and robustly induced in leaves by saline-alkali and oxidative cues, such as NaCl, Na_2_CO_3_, NaHCO_3_, and H_2_O_2_, with transcript accumulation peaking around 12 h, consistent with an early stress-responsive node [39]. Overexpression of *LpGDSL* confers marked saline-alkali tolerance, manifested by improved growth, sustained chlorophyll content, and reduced cellular damage under stress [39]. Mechanistically, *LpGDSL* enhances lignin deposition and helps maintain reactive oxygen species (ROS) homeostasis by balancing O_2_^−^ and H_2_O_2_ accumulation, which is associated with diminished oxidative injury to membranes and cell walls. Protein interaction and promoter analyses further place *LpGDSL* within a defined regulatory module: it physically interacts with the *LpBCP* and is transcriptionally activated by B3 transcription factors binding its promoter, thereby linking a GELP-mediated lipid process to lignin biosynthesis, ROS detoxification, and transcriptional control [39]. In the context of GELPs, which are still most often characterized mainly by expression patterns or single stress phenotypes [1,14,37], this multilayered functional dissection of *LpGDSL* remains exceptional within the family (Figure 1).

Pan-angiosperm phylogenetic analyses have resolved GELPs into multiple orthogroups and subfamilies with distinct duplication histories and lineage-specific expansions, particularly in large-genome crops [1,7,51]. In *G. max*, *G. hirsutum*, *S. alfredii*, *B. napus*, and other species, genome-wide inventories consistently reveal 80–400 GELP loci, with a substantial fraction showing inducible or tissue-specific expression under salt, drought, osmotic, temperature, and heavy metal stresses [19]. Functional work on individual genes, such as *GmGELP28* in *G. max* and *SaGLIP8* in *S. alfredii*, demonstrates that overexpression can enhance tolerance to salinity, drought, or cadmium, often coupled with changes in ROS-scavenging capacity, ion homeostasis, or lipid mobilization [2]. Collectively, these studies support a unifying view of GELPs as modulators of membrane and cell-wall interfaces, embedded in hormone and ROS-responsive regulatory networks.

Within this broader framework, stress-responsive cytochrome P450 genes, such as *GsCYP93D1* from wild *G. max*, provide a complementary strategy to enhance tolerance. By reinforcing antioxidant defenses and activating alkaline, ABA-responsive marker genes, such as *GsCYP93D1*, strengthen ROS detoxification and stress signaling [38,52]. Although studies on *GsCYP93D1* do not characterize GELP genes or their enzymatic activities and thus cannot directly define GELP-specific mechanisms, they underscore a general principle that robust stress resilience emerges from coordinated control of ROS homeostasis, hormone pathways and membrane-linked metabolism, the same interconnected processes in which GELPs are increasingly implicated [20,26,53].

From a breeding standpoint, GELPs that reproducibly enhance tolerance to drought, salinity, freezing, heavy metals, or combined stresses are attractive candidates for marker development, allele mining in diverse germplasm, and precision editing. The availability of orthogroup-based phylogenies and expression atlases enables prioritization of GELP clades with conserved stress-responsive functions across species, thereby supporting rational transfer of candidate genes between model plants and crops. Stress-inducible promoters of GELP genes may also be repurposed to drive protective transgenes in a temporally and spatially controlled manner, minimizing fitness penalties under non-stress conditions.

A central frontier for the field, therefore, is to move from correlative genomics and expression surveys to mechanistic and systems-level dissection. Despite extensive inventories, only a small proportion of GELPs have been functionally characterized in planta, and even fewer have defined substrates, interaction partners, or signaling outputs. High-priority efforts should integrate targeted genome editing, including higher-order mutants and gain-of-function lines with lipidomics, metabolomics, and cell-type-resolved transcriptomics under well-controlled stress regimes. Applied across representative orthogroups and crop species, such strategies will clarify how discrete GELP nodes remodel lipid landscapes, coordinate with ROS and hormone pathways, and ultimately shape stress resilience, creating a mechanistic foundation for rational deployment of GELPs in future climate-resilient breeding programs. In parallel, systematic phenotyping of natural and induced GELP variants in multi-environment trials will be essential to quantify their effects on yield stability, quality, and disease resistance, and to integrate GELP markers into genomic selection and other modern breeding frameworks.

## 5. Methodological and Multi-Omics Approaches to Study GELP Structure and Function

### 5.1. Genome-Wide Identification and Transcriptomic Profiling

Systematic mining of plant genomes has defined GELP family size, exon-intron organization and duplication history across many crops, for instance, *G. hirsutum* contains 389 GELPs [7]; *G. max* contains 194 GELPs [2]; *D. villosum* holds 193 GELPs [14], *B. rapa* contains 121 GELPs [15], *V. vinifera* contains 83 GELPs [25], *Dendrobium catenatum* holds 52 GELPs [4], *R. communis* contains 96 GELPs [19], *Cajanus cajan* contains 112 GELPs [54] and *A. thaliana* contains 105 GELPs. Family-wide surveys in *G. max* and *Gossypium spp* have identified 194 and 389 GELPs, respectively, revealed that whole-genome/segmental and tandem duplications are major drivers of expansion, and showed that most paralogs retain conserved five-exon architectures under purifying selection while diverging in regulatory profiles [7,35].

RNA-seq expression atlases across organs, development, and treatments are widely used to infer tissue- and stress-specific GELP roles. In *G. hirsutum*, clustering of transcriptomes from multiple tissues and stress conditions revealed GELP subgroups specialized for stamens, leaves, stems, and fiber development, as well as diverse abiotic and biotic stress responses [7,15]. *G. max* RNA-seq and RT-qPCR defined subsets induced by drought, salinity, and ABA, leading to functional validation of *GmGELP28* in drought and salt tolerance [35]. Similar genome-wide expression profiling has been conducted under high temperature and salt stress in *R. communis* [19], and during barley stripe mosaic virus infection in *D. villosum* [14]. Additional RNA-seq datasets for berry ripening and H_2_O_2_ treatment in *V. vinifera* [25], and for seed germination and cadmium stress in *S. alfredii* [37] further highlight stress-responsive GELP candidates. Together, these studies illustrate how transcriptomics can be used to prioritize GELPs for functional characterization.

### 5.2. Multi-Omics Integration to Link GELPs with Metabolism and Regulation

Multi-omics strategies are increasingly applied to connect GELP activity with specific metabolic and regulatory outputs. Lipidomics has been used to track changes in defined lipid species in anthers and seeds, while metabolomics captures broader shifts in secondary metabolites associated with GELP perturbations, as shown for anther-expressed GELPs controlling *O. sativa* reproductive development and seed-specific GELPs such as *BnSCE3* and *BnLIP2* in *B. napus*, as well as *SaGLIP8* in *S. alfredii* under cadmium stress [2,53]. In contrast, proteomics-based studies of GELPs remain scarce, and most datasets still lack direct information on GELP protein abundance, post-translational modification, or interaction partners. Co-expression clustering and weighted gene co-expression network analysis (WGCNA) applied to these datasets further identify modules and hub GELPs that are specifically induced by abiotic or biotic stresses and hormones, paralleling network-level analyses of drought and cold responses in *A. thaliana* and *G. hirsutum* [55,56].

**Figure 2:**
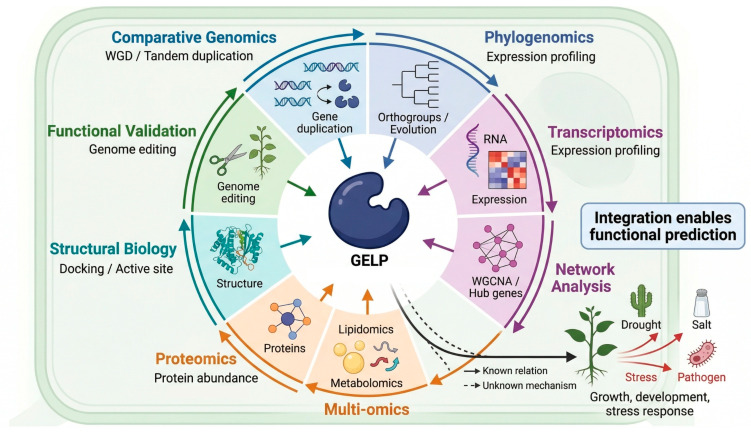
Integrative multi-omics framework for functional characterization of plant GDSL esterase/lipase (GELP) proteins. This schematic presents a conceptual workflow that integrates comparative genomics, phylogenomics, transcriptomics, co-expression network analysis, metabolomics, lipidomics, proteomics, structural modeling, and functional validation to elucidate GELP evolution and biological functions and to identify key regulators for crop improvement. This figure is generated using BioRender.com.

Integrated multi-omics and systems-genetics approaches represent an important frontier. Combining targeted genome editing, including higher-order GELP mutants, with lipidomics, metabolomics, and cell-type and single-cell-resolved transcriptomics and chromatin-accessibility profiling under controlled stress regimes is needed to mechanistically resolve how individual GELPs remodel lipid landscapes, coordinate with ROS and hormone pathways, and influence yield, quality, and stress resilience [9,57,58]. Integrating these datasets with gene-duplication-aware comparative frameworks, such as integrative gene-duplication and genome-wide analysis, will be essential to prioritize lineage-specific GELP duplicates, build predictive models of GELP-centered regulatory networks, and translate comparative and functional genomics into actionable targets for crop improvement (Figure 2).

## 6. Conclusions and Future Perspectives

Plant GELPs have emerged as important modulators and contributors to lipid metabolism, development, and stress resilience in land plants. The GELP family is diverse yet conserved in land plants; its isozymes differ in activity, substrate specificity, and product formation, reflecting extensive expansion and structural diversification documented in *A. thaliana*, *O. sativa*, *G. max*, *G. hirsutum*, *B. napus*, and other species. GELPs and their hydrolyzing products contribute to cuticle and secondary wall formation, seed lipid mobilization, reproductive development, and hormone-mediated responses to biotic and abiotic stresses. Over the past two decades, genome-wide and comparative analyses have cataloged large GELP repertoires in numerous crops and model plants, and a growing subset has been functionally characterized, particularly in *A. thaliana*, *O. sativa*, *Solanum lycopersicum* (*S. lycopersicum*), *G. max*, *B. napus*, and *G. hirsutum*. Nevertheless, for most family members, the in planta functions, enzymatic mechanisms, and regulatory networks remain poorly defined, such that current knowledge still falls short of what is required to support sustainable agriculture under rapidly changing climates. Natural variation in GELP coding and regulatory sequences, revealed by population genomics and landscape-scale studies, offers a rich source of alleles for probing their physiological and evolutionary importance and for enhancing adaptation to drought, salinity, heat, and pathogens. Recent classification of GELPs into orthogroups across flowering species and genome-wide work in *G. hirsutum*, *B. napus*, *R. communis*, and moss provide a framework to link sequence variation to function and environmental adaptation.

From a biotechnological and breeding perspective, GELPs occupy several strategic “control points” in plant biology. At the reproductive level, GELP genes that determine male fertility and pollen-stigma interactions can be mobilized to generate robust hybrid seed production systems. In seeds and fruits, GELPs influencing size, surface properties, composition, and anti-nutritional factors offer levers to improve yield, nutritional quality, and postharvest traits. In vegetative tissues and at the cell wall–cuticle interface, stress-responsive GELPs that modulate lipid signaling, cuticle integrity, lignification, and ROS balance constitute promising targets for enhancing multi-stress resilience without severely compromising growth.

To realize this potential, future work should perform systematic allele mining and association mapping of GELP loci across diverse germplasm panels to identify variants linked to yield, quality and stress-tolerance traits; deploy CRISPR/Cas-based multiplex editing and base editing to generate fine-tuned GELP allelic series, enabling detailed genotype-phenotype dissection; integrate GELP markers into genomic selection and marker-assisted backcrossing schemes; and explore GELP enzymes as versatile biocatalysts in metabolic engineering and synthetic biology for the production of high-value metabolites, green chemicals and novel agrochemicals.

To fully elucidate GELP functions in plant development, lipid metabolism and stress responses, and to exploit them as targets for breeding and engineering resilient crops, future work should integrate comparative and functional genomics with reverse and forward genetics, biochemistry, multi-omics, and gene regulatory network approaches, while systematically assessing the effects of single and combined GELP mutations on yield, quality, and stress tolerance. Bridging mechanistic enzymology with field-scale performance data will ultimately determine how effectively GELP-centered strategies can contribute to climate-resilient, high-productivity agriculture.

## Figures and Tables

**Table 1:** Reported function of GELPs in different plant species.

Functional Category (Development/Stress)	Species	GELP Genes	Key Role	Potential Application	References
Male fertility and hybrid seed production	*O. sativa*; *Z. mays*; *T. aestivum*; *A. thaliana*	*OsGELP34*, *OsGELP110*, *OsGELP115*; *ZmMs30*; *TaGELP073*; *GELP77*	Essential for another cuticle integrity, pollen exine formation, and male fertility	Development of stable male-sterile and restorer lines for hybrid breeding; fine-tuning of fertility under stress	[32,33,34,35]
Seed size, composition, and anti-nutritional factors	*B. napus*; *G. hirsutum*	*BnSCE3*, *BnLIP2*; *GhGLIP*	Reduced seed sinapine, increased choline; larger seeds with higher fresh weight and storage reserves	Improved oil and protein quality; reduced anti-nutrients in oilseeds via marker-assisted selection or genome editing	[7,17,27]
Fiber and vegetative growth traits	*Gossypium barbadense*	*GbGELP*	Controls fiber elongation and micronaire; affects root and shoot growth in *A. thaliana*	Enhancement of fiber quality and biomass, integration in QTL-based breeding for *G. hirsutum* improvement	[36]
Abiotic stress tolerance (salt, drought, heavy metal, freezing)	*G. max*; *S. alfredii*; *L. pumilum*; *R. communis*	*GmGELP28*; *SaGLIP8*; *LpGDSL*; *RcGELPs*	Overexpression enhances tolerance to salinity, drought, cadmium, or saline–alkali stress, often via ROS homeostasis and cell wall strengthening.	Introgression or editing of GELP alleles to confer multi-stress tolerance; development of stress-inducible GELP expression cassettes for transgenic crops	[2,19,37,38,39]
Pathogen and virus interaction	*D. villosum*; *C. lanatus*	*DvGELP53*; *ClGELPs*	Involved in long-distance movement of BSMV; associated with gummy stem blight resistance	Use in breeding for disease resistance and in designing virus-resistance strategies.	[3,11,14]

## Data Availability

No new data were generated in the manuscript.

## Abbreviations

The following abbreviations are used in this manuscript:

ABA	abscisic acid
ACh	acetylcholine
AChE	acetylcholinesterase
BSMV	barley stripe mosaic virus
ChE	cholinesterase
*CUS1*	cutin synthase 1
ET	ethylene
GA	gibberellin
GDSL	glycine–aspartate–serine–leucine motif (GDSxxDxG) lipase family
GELPs	GDSL esterase/lipase
*GRP17*	glycine-rich protein 17
Ka/Ks	ratio of nonsynonymous to synonymous substitution rates
LAE	lanatoside-15′-O-acetylesterase
RNA-seq	RNA sequencing
*RGE1*	retarded growth of embryo1
ROS	reactive oxygen species
RT-qPCR	reverse-transcription quantitative PCR
SA	salicylic acid
SGNH	serine–glycine–asparagine–histidine hydrolase superfamily
SD-RLK28	stigma determinant receptor-like kinase 28
WGCNA	weighted gene co-expression network analysis
H_2_O_2_	hydrogen peroxide
ER	endoplasmic reticulum
JA	jasmonic acid
QTL	quantitative trait loci
bHLH	basic helix–loop–helix
NaCl	sodium chloride
NaHCO_3_	sodium bicarbonate
